# Vesicular LL-37 Contributes to Inflammation of the Lesional Skin of Palmoplantar Pustulosis

**DOI:** 10.1371/journal.pone.0110677

**Published:** 2014-10-16

**Authors:** Masamoto Murakami, Takaaki Kaneko, Teruaki Nakatsuji, Kenji Kameda, Hidenori Okazaki, Xiuju Dai, Yasushi Hanakawa, Mikiko Tohyama, Akemi Ishida-Yamamoto, Koji Sayama

**Affiliations:** 1 Department of Dermatology, Ehime University Graduate School of Medicine, Ehime, Japan; 2 Department of Dermatology, Asahikawa Medical College, Asahikawa, Japan; 3 Division of Dermatology, University of California San Diego, and VA San Diego Healthcare Center, San Diego, California, United States of America; 4 Integrated Center for Science, Ehime University Graduate School of Medicine, Ehime, Japan; University of Leuven, Rega Institute, Belgium

## Abstract

“Pustulosis palmaris et plantaris”, or palmoplantar pustulosis (PPP), is a chronic pustular dermatitis characterized by intraepidermal palmoplantar pustules. Although early stage vesicles (preceding the pustular phase) formed in the acrosyringium contain the antimicrobial peptides cathelicidin (hCAP-18/LL-37) and dermcidin, the details of hCAP-18/LL-37 expression in such vesicles remain unclear. The principal aim of the present study was to clarify the manner of hCAP-18/LL-37 expression in PPP vesicles and to determine whether this material contributed to subsequent inflammation of lesional skin. PPP vesicle fluid (PPP-VF) induced the expression of mRNAs encoding IL-17C, IL-8, IL-1α, and IL-1β in living skin equivalents, but the level of only IL-8 mRNA decreased significantly upon stimulation of PPP vesicle with depletion of endogenous hCAP-18/LL-37 by affinity chromatography (dep-PPP-VF). Semi-quantitative dot-blot analysis revealed higher concentrations of hCAP-18/LL-37 in PPP-VF compared to healthy sweat (2.87±0.93 µM vs. 0.09±0.09 µM). This concentration of hCAP-18/LL-37 in PPP-VF could upregulate expression of IL-17C, IL-8, IL-1α, and IL-1β at both the mRNA and protein levels. Recombinant hCAP-18 was incubated with dep-PPP-VF. Proteinase 3, which converts hCAP-18 to the active form (LL-37), was present in PPP-VF. Histopathological and immunohistochemical examination revealed that early stage vesicles contained many mononuclear cells but no polymorphonuclear cells, and the mononuclear cells were CD68-positive. The epidermis surrounding the vesicle expresses monocyte chemotactic chemokine, CCL2. In conclusion, PPP-VF contains the proteinase required for LL-37 processing and also may directly upregulate IL-8 in lesional keratinocytes, in turn contributing to the subsequent inflammation of PPP lesional skin.

## Introduction

“Pustulosis palmaris et plantaris”, or palmoplantar pustulosis (PPP), is a chronic pustular dermatitis characterized by intraepidermal palmoplantar pustules [1]. On careful observation in the clinic, a PPP lesion exhibits several unique characteristics including vesicles, pustules, erythema, lichenification, and abnormal desquamation. Although PPP is a common skin disease that is often recalcitrant to available treatments, the pathogenesis of the condition remains unknown. Prior to pustule formation, vesicles form early in the acrosyringium, and antimicrobial peptides found in human sweat, hCAP-18/LL-37 and dermcidin are present in vesicles of the palms and soles [2]. In eccrine sweat, these components protect the body surface via the innate immune system. Dermcidin is continuously secreted in eccrine sweat but is not induced during inflammation [3], [4]. In contrast, hCAP-18/LL-37 is induced in inflammatory conditions such as psoriasis and wound healing [5], [6].

Later, secondary leukocyte accumulation in vesicles is associated with expression of complement and/or IL-8 in the stratum corneum or the surrounding epidermal keratinocytes [7]. Furthermore, interleukin (IL)-17-positive cells infiltrate around the acrosyringium [8]. Although the mechanism of abnormal desquamation remains unclear, aberrant expression of kallikrein-related peptidases (KLK-5, -7, and -14) in lesional skin may be important in this context [9].

Human skin contains two major classes of antimicrobial peptides: the cathelicidins [10]–[12] and the β-defensins [13]–[15]. Like many other antimicrobial peptides, cathelicidins are synthesized as preproproteins [12]. The only human cathelicidin is hCAP-18 [5], [16], expressed in leukocytes and on a variety of epithelial surfaces. hCAP-18 is processed by a proteinase, principally proteinase-3, to the mature form, LL-37, which exhibits antimicrobial activity [17]. hCAP-18/LL-37 has been detected in human keratinocytes, but only at sites of inflammation, suggesting that the peptide functions primarily in response to injury. Though main role of LL-37 is “antibacterial” but several studies reported that LL-37 is chemotactic in vitro, inducing selective migration of human peripheral blood monocytes, neutrophils, and CD4-positive T cells [18], [19]. Recent evidence indicates that skin antimicrobial peptides, including cathelicidin, are chemotactic for PMNs [20]. LL-37, the mature form of cathelicidin, plays an important role in skin barrier function and contributes to inflammation of skin lesions [21]–. In addition, LL-37 can be processed to physiological fragments such as RK-31, KR-30, and KS-20, after secretion in sweat. They exhibit antimicrobial activity as LL-37 shows [24] However, several additional LL-37 fragments are found in the pathogenesis of rosacea, one the inflammatory skin disorders, and they contribute the inflammatory cytokines up-regulations [25]. Hence, LL-37 regarded as a double-edged sword for skin defense barrier and regeneration.

We have observed that lesions do not develop pustules or scales if vesicle/pustule ruptures occur, suggesting that the vesicle/pustule contains some heretofore-undefined factor causing subsequent inflammation. As mentioned above, hCAP-18/LL-37 occurs in PPP vesicles, and may be the factor triggering inflammatory changes.

In the present study, we sought to detail the manner of hCAP-18/LL-37 expression in PPP vesicular fluid (PPP-VF) and to determine whether this material contributed to subsequent inflammation of lesional skin.

## Materials and Methods

### Ethics Statement

All procedures that involved human subjects except the skin biopsy received prior approval from the Ethics Committees of Asahikawa Medical College and Ehime University Graduate School of Medicine. The skin biopsy procedure from the patient was approved by the Ethics Committee of Asahikawa Medical University. We have already got an approval by the Ethics Committee of Ehime University and confirmation of written informed consent from the donor’s patient for the collection and generation of the cell lines described [20]. This study was conducted according to the principles of the Declaration of Helsinki. All subjects provided written informed consent.

### PPP vesicle and sweat collection

Fifteen volunteers (13 females and 2 males; mean age: 62.7±18.5 years, range: 33–82 years) with 2–10-year histories of PPP were recruited. Additional clinical information at the first visit to our hospital is as follows; smoking history (10/15, over 20 yrs), sternoclavicular joint pain (3/15), anti-streptlysin O test (5/15), periodontitis (3/15). All subjects had used only topical steroid ointments and had not been treated previously with any systemic therapy. Two board-certified dermatologists performed clinical diagnoses. To obtain early stage material, lesional vesicles were carefully observed using a dermatoscope and samples were collected as follows: The skin lesions were cleaned with 70% (v/v) alcohol; lids were removed using 18-G needles; and vesicle fluid was collected immediately (using a micropipette) from several small vesicles. About 3 µl of vesicle fluid were diluted in 30-µl double-distilled water (DDW) and stored in a microtube at –80°C prior to evaluation. Healthy sweat served as a control. After 30 min of exercise, eccrine sweat was collected from the forearms of 14 healthy volunteers (students of Ehime University; three females and eight males; age, 19–23 years) using tissue paper, as described previously [24]. After collection, crude sweat was centrifuged at 17,000×g for 10 min, and the supernatants collected and stored at –80°C prior to use.

### Tissue sampling

Punch biopsies (∼5 mm in diameter) were taken from the palmar vesicular lesions of five PPP cases at Asahikawa Medical University with their written informed consent, and subjected to pathological diagnosis at our clinic. Specimens were fixed in 10% (v/v) buffered formalin overnight and next embedded in paraffin blocks for routine pathological diagnosis. Sections 4 µm in thickness were prepared for hematoxylin-and-eosin (H&E) staining and immunostaining.

### Cell cultures and stimulation

Primary normal human keratinocytes (NHKs) were isolated from surgically discarded neonatal skin samples (dactylosymphysis, 0 M) and cultured in MCDB153 medium supplemented with insulin (5 µg/ml), hydrocortisone (5×10^−7^ M), ethanolamine (0.1 mM), phosphoethanolamine (0.1 mM), bovine pituitary extract (50 mg/ml), and Ca^2+^ (0.03 mM), as described previously [26]. Subconfluent keratinocyte cultures that had been passaged four times were used in stimulation experiments. Cells were incubated with 3 µM synthetic LL-37 peptide (this level was shown to be appropriate in a pilot study) for various times (0, 2, 4, 8, 20, and 24 h) at 37°C.

### Preparation of human living skin equivalents (LSEs) and stimulation thereof

The LSE preparation method has been described previously [20], [27]. Briefly, a collagen gel was prepared by mixing 6 volumes of ice-cold porcine collagen type I solution (Nitta Gelatin, Osaka, Japan) with 1 volume of 8× DMEM (Gibco, Auckland, NZ), 10 volumes of 1× Dulbecco’s Minimal Essential Eagle’s Medium (DMEM) supplemented with 20% (v/v) FCS, and 1 volume of 0.1 M NaOH. The final collagen concentration was 0.8 mg/ml. One milliliter amounts of the mixture were added to culture inserts (Transwel-COL, membrane pore size 3 µm; Costar, Corning, NY, NY) in a six-well Costar culture plate (Corning). After gel polymerization at 37°C, two volumes of a fibroblast suspension (5×10^5^ cells/ml in 1× DMEM supplemented with 10% [v/v] FCS) were added to eight volumes of collagen solution, and 3.5 ml of the mixture were applied to each insert. When the fibroblast-containing gel had polymerized, DMEM supplemented with 10% (v/v) FCS and ascorbic acid (50 ng/ml final concentration) was added. The culture medium was changed twice weekly. Five days after dermal components were prepared, 6.0×10^5^ keratinocytes in 60-µl MCDB 153 type II medium were seeded onto the concave surfaces of contracted gels. The keratinocytes were kept submerged in culture medium for 2 days. When the keratinocytes attained confluence, the LSE was lifted to form an air-liquid interface and cornification medium [27] was added. This medium was changed every other day. Ten days after airlift, the LSEs were used in PPP vesicle stimulation experiments. At the end of each experiment, LSEs were fixed in 20% (v/v) formalin and embedded in paraffin for histological evaluation of morphology.

To stimulate LSEs with PPP-VF, 10 µl PPP-VF was diluted in 100 µl 1% (w/v) agarose gel (SeaPlaque agarose; FMC Bioproducts, Rockland, ME). The heat-melted gel was placed into the cap of a 1.5-ml tube and cooled to RT. Next, a gel cylinder was excised using a disposable biopsy punch (6 mm in diameter, Kai Medical, Kyoto, Japan) and placed on the surface of an LSE, followed by incubation for 12 h at 37°C in a humidified incubator under 5% (v/v) CO_2_. As a control, 100 µl of a 1% (w/v) agarose gel cylinder containing 10 µl of eccrine sweat was prepared. After depletion of endogenous hCAP-18/LL-37 in PPP-VF (as described below), 10-µl amounts of such depleted PPP-VF diluted in 100 µl of 1% (w/v) agarose gel were incubated with LSEs.

### Synthetic LL-37 and native human proteinase 3 peptides

Both authentic and scrambled LL-37 were commercial preparations (Peptide Institute Inc., Osaka, Japan; and Eurogentec, Seraing, Belgium, respectively). Peptides were purified to over 95% by HPLC and their identities confirmed by mass spectrometry as described previously [2]. Native human proteinase 3 was purchased from Cell Sciences (Canton, MA).

### Preparation of the hCAP-18 plasmid

A PCR product encoding full-length hCAP-18 was generated using cDNA obtained from NHKs stimulated with vitamin D; the forward PCR primer was (5′-TAAGGCCTCTGTCGACCAGGTCCTCAGCTACAAGGAAGC-3′) and the reverse primer (5′-CAGAATTCGCAAGCTTCTAGGACTCTGTCCTGGGTACAAG-3′); both primers contained restriction enzyme sites. Amplified DNA was digested with *Sal*I and *Hind*III, inserted into the In-Fusion Ready pEcoli-Nterm 6×HN vector (Clontech Laboratories, Mountain View, CA), and transformed into competent cells [*Escherichia coli* BL21 (DE3); Invitrogen, Carlsbad, CA].

### Preparation of recombinant hCAP-18 (rhCAP-18) with a GST tag

The rhCAP-18 peptide was prepared as full-length human cathelicidin using a cell-free protein synthesis system employing wheat germ ribosomal RNA [28], [29]. Briefly, the hCAP-18 cDNA was inserted into a pEUE01-GST-N2 expression vector containing a GST tag region. GST-hCAP-18 proteins were automatically synthesized using a Robotic Protein Synthesizer Protemist DT II (CellFree Sciences, Matsuyama, Japan). The transcription mixture (250 µl) containing 25 µg plasmid DNA, 80 mM HEPES-KOH (pH 7.8), 16 mM magnesium acetate, 2 mM spermidine, 10 mM dithiothreitol, 2.5 mM each of the four nucleoside triphosphates, 250 U of SP6 RNA polymerase (Promega, Madison, WI), and 250 U RNasin (Promega), was incubated for 6 h at 37°C. Next, the solution was mixed with 250-µl wheat germ extract WEPRO7240G (CellFree Sciences) supplemented with 1 µl of 20 mg/ml creatine kinase in a single well of a six-well plate. The substrate mixture (5.5 ml; 30 mM HEPES-KOH [pH 7.8], 100 mM potassium acetate, 2.7 mM magnesium acetate, 0.4 mM spermidine, 2.5 mM dithiothreitol, 0.3 mM amino acid mix, 1.2 mM ATP, 0.25 mM GTP and 16 mM creatine phosphate [CellFree Sciences]) was placed on top of the translation mix and incubated at 17°C for 20 h. Synthetic GST-rhCAP-18 was purified on a glutathione Sepharose 4B column (GE Healthcare, Uppsala, Sweden). The protein solution was loaded onto the column and the column next washed with PBS. We sought to remove the GST tag from the recombinant peptide using Turbo-TEV protease (GST fusion) employing a standard procedure, but the peptide remained attached to the resin and could not be eluted. Therefore, we used the GST-rhCAP-18 peptide in processing experiments.

### Antibodies

A mouse anti-LL-37 monoclonal antibody was purchased from Santa Cruz Biotechnology, Inc. (Santa Cruz, CA; catalog no. Sc-166770). Chicken polyclonal antibodies against the cathelin-domain (CATH) peptides were produced by Aves Labs (Tigard, OR), as described [30]. Mouse anti-PR3 monoclonal antibody, mouse anti-CD68 (KP1) antibody, and mouse-anti MCP1 (CCL2) antibody were purchased from Abcam (Tokyo, Japan) (catalog nos. ab91181, ab955, and ab9669, respectively). Mouse anti-CD56 (1B6) antibody was purchased from Nichirei Biosciences Inc. (Tokyo, Japan).

### Antibody column depletion of endogenous hCAP-18/LL-37 in PPP-VF (dep-PPP-VF)

To deplete endogenous vesicular hCAP-18/LL-37, an anti-hCAP-18/LL-37 antibody column was prepared using the anti-LL-37 monoclonal antibody and a Protein G HP SpinTrap/Ad Spin Trap (GE Healthcare Life Sciences, Tokyo, Japan) according to the manufacturer’s instructions. Ten microliter amounts of PPP-VF were diluted in 50 µl of DDW, and endogenous hCAP-18/LL-37 removed by affinity chromatography, as confirmed by Western blotting (described below).

### Processing experiment using depleted PPP vesicles

After preparing dep-PPP-VF as described above, 20-µl amounts of depleted solution were immediately applied to GST-rhCAP-18-Sepharose columns. The column resin was incubated with dep-PPP-VF, eccrine sweat, or without any addition, at 37°C for 6 h. All samples were subsequently centrifuged at 1,000 rpm for 15 s; eluent (flowthrough) and resin collected; and 5-µl amounts of both eluent and resin boiled in Laemmli sample buffer and subjected to SDS-PAGE gel electrophoresis as described below. The experiments were repeated three times.

### Dot-blot analysis and densitometry

To estimate the concentrations of hCAP-18/LL-37 in sweat and PPP-VF, we performed semi-quantitative dot-blot analysis. Briefly, 10-µl PPP-VF was diluted in 100-µl DDW and spotted onto nitrocellulose membranes containing serial dilutions of the LL-37 synthetic peptide using a Bio-Dot apparatus (Bio-Rad Laboratories, Hercules, CA). As a negative control, 10 µM of scrambled LL-37 peptide was applied to a membrane. After air-drying, membranes was treated with blocking solution (0.1% TTBS: 5% [w/v] nonfat milk in 0.1% [v/v] Tween 20/Tris-buffered saline [TBS] with 150 mM NaCl, and 10 mM Tris Base [pH 7.6]) for 60 min at room temperature (RT), and mouse anti-LL37 monoclonal antibody (1∶100 in blocking solution) was next added, followed by overnight incubation at 4°C. After three washes with 0.1% (v/v) TTBS, the membranes were incubated with horseradish peroxidase (HRP)-labeled rabbit anti-mouse IgG polyclonal antibody (1∶2,000 in blocking solution, Bio-Rad) for 60 min at RT. After washing with 0.1% TTBS, membranes were immersed in ECL solution (Western Lightning Chemiluminescence Reagents Plus; GE Healthcare, Buckinghamshire, UK) for 60 s and visualized using the LAS-4000 imaging system (GE Healthcare) according to the manufacturer’s instructions. To estimate hCAP-18/LL-37 concentrations, the intensity of dot staining was compared to that of a standard curve constructed by spotting concentrations of the synthetic peptide onto the nitrocellulose membrane. Dot staining intensities were measured in triplicate experiments using NIH-image software, as described previously [9].

### Western blotting

Proteins in PPP-VF (10 µl), and in GST-hCAP18 recombinant protein preparations after incubation with various factors, were resolved on 15% (w/v) Tris-HCl gels (BIO CRAFT, Tokyo, Japan) and transferred to PVDF membranes (Immobilon-P; Millipore, Darmstadt, Germany); 3.2 pmol of the LL-37 synthetic peptide served as a positive control. Membrane bands were visualized as described above. To confirm the identities of bands detected by Western blotting, the filter was stripped of antibody using WB Stripping Solution (Nacalai Tesque, Kyoto, Japan) following the manufacturer’s instructions, and next reacted with a chicken anti-cathelin polyclonal antibody (1∶2,000) and an HRP-conjugated-anti-chicken IgY goat antibody (1∶2,000, Promega), as described previously [30].

To detect proteinase 3 in PPP-VF, 10-µl PPP-VF were subjected to 15% (w/v) Tris-HCl gel electrophoresis using native human proteinase 3 as a positive control. A mouse anti-proteinase 3 monoclonal antibody (1∶30) was used as primary antibody to detect the protein, as described above.

### Immunohistochemistry

Tissue sections were immersed in PBS after deparaffinization and endogenous peroxidase activity was blocked by incubation with 0.3% (v/v) H_2_O_2_ in methanol for 30 min. After washing with phosphate buffered saline (PBS), immunostaining with mouse anti-CD68 (1∶200) and mouse anti-CD56 antibodies (ready-to-use) was performed using a Histofine SAB-PO kit (Nichirei Biosciences Inc.) according to the manufacturer’s instructions. As a negative control, the polyclonal and monoclonal antibodies were replaced by normal mouse preimmune IgG diluted with PBS containing 3% (w/v) BSA to the protein concentrations of the primary antibodies. Nuclear counterstaining was performed using hematoxylin. All procedures were conducted at RT, except for the initial antibody incubation (4°C, overnight).

### Enzyme-linked immunosorbent assays (ELISAs)

NHK monolayers were treated with 3 µM of an LL37 synthetic peptide (which was shown to be appropriate in a pilot experiment) at 37°C for 12 h, and the culture supernatants collected to measure protein expression levels. ELISA kits detecting IL-1α, IL-1β, IL-8, and IL-17C were purchased from R&D Systems (Minneapolis, MN) and used according to the manufacturer’s protocols. Optical densities at 450 nm were measured using an Immuno Mini NJ-2300 microplate reader. All assays were performed in triplicate.

### RNA preparation and quantitative real-time PCR (qRT-PCR)

Total RNA was isolated using Isogen (Nippon Gene, Tokyo, Japan) and cDNA was prepared from 1-µg amounts of total RNA using the iScript cDNA synthesis kit (Bio-Rad Laboratories) according to the manufacturer’s instructions. TaqMan probes for glyceraldehyde-3-phosphate dehydrogenase (GAPDH), IL-1α, IL-1β, IL-8, IL-22, and IL-17 (A, F, and C) were obtained from Applied Biosystems (Foster city, CA). qRT-PCR was performed on an ABI PRISM 7700 sequencing platform (Applied Biosystems) according to the manufacturer’s instructions. The mRNA levels of target genes were normalized to that of GAPDH. Gene expression levels in treated cells were compared to those in untreated cells. Data were analyzed using the Comparative Ct Method, where Ct indicates the number of cycles required to reach an arbitrary threshold [31].

### Statistical analysis

Quantitative data are expressed as means *±* standard deviations. The data from dot-blot analysis and real-time PCR were evaluated using the STATFLEX software (version 6.0, ARTEC Inc., Osaka, Japan). The paired Student’s *t*-test was used to compare between-group differences, and *p*-values<0.05 were considered to indicate statistical significance.

## Results

### PPP-VF induces inflammatory cytokine mRNA expression in LSEs

**Figure 1:**
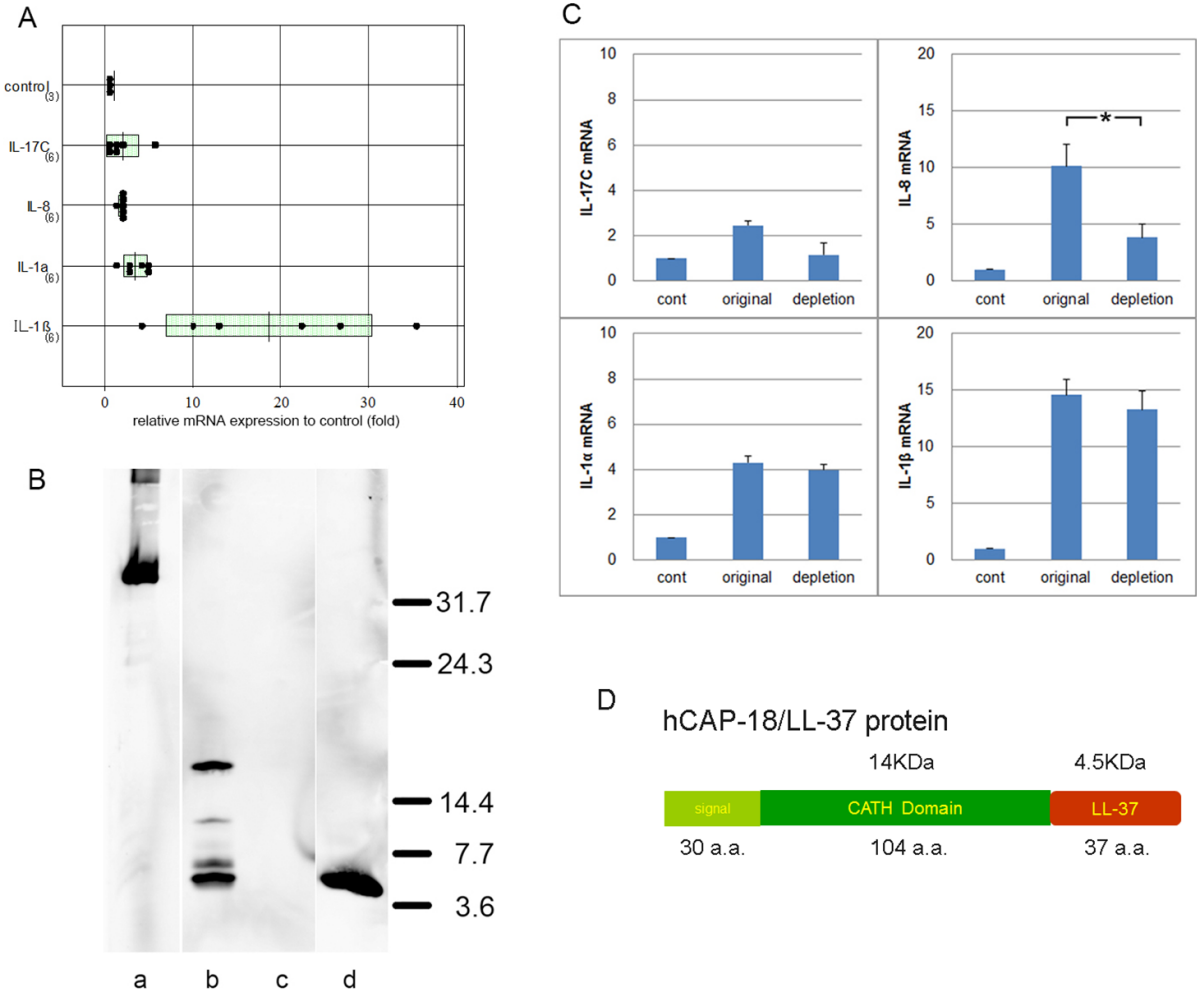
PPP vesicles stimulate expression of cytokine-encoding mRNAs in LSEs. PPP vesicles were suspended in 1% (w/v) agar and mRNAs measured using qRT-PCR. **A)** All of IL-17C (2.00±1.79-fold), IL-8 (1.8±1.7-fold), IL-1α (3.47±1.28-fold), and IL-1β (18.67±11.72-fold) were upregulated compared to the levels in non-treated LSEs (controls). The levels of cathelicidin, IL-8, IL-1α, and IL-1β differed significantly from those in control sweat. **B)** Western blotting showed that the hCAP-18/LL-37-depleted PPP-VF sample contained bands equivalent to hCAP-18 (18 kDa; full-length); an intermediate-sized fragment (∼14 kDa); mature LL-37 (4.5 kDa); and two additional bands (lane b). Lane a: the GST-hCAP18 peptide prior to incubation; Lane b: PPP-VF before depletion; Lane c: PPP-VF after depletion of endogenous hCAP-18/LL-37; Lane d: synthetic LL-37 peptide (3.2 pmol). **C)** mRNA expression levels in LSEs stimulated by original and depleted PPP-VF, as calculated via qRT-PCR. *p<0.05. **D)** The illustration of structure of hCAP-18/LL-37.

After incubation with PPP-VF, the expression levels of cytokine mRNAs in LSEs were evaluated via qRT-PCR (Fig. 1A). All of IL-17C (2.00±1.79-fold), IL-8 (1.8±1.7-fold), IL-1α (3.47±1.28-fold), and IL-1β (18.67±11.72-fold) were upregulated compared to non-treated LSEs (control). None of IL-22, IL-17A, or IL-17F was detected. For IL-8, IL-1α, and IL-1β, the increases were statistically significant (Fig. 1A).

### Confirmation of LL-37 expression in PPP-VF and preparation of depleted PPP-VF using an anti-LL-37 antibody column

Expression of hCAP-18 in PPP vesicles was confirmed using Western blotting. hCAP-18 (18 kDa; full-length), an intermediate-sized fragment (∼14 kDa), and mature LL-37 (4.5 kDa), were detected, as were additional fragments of ∼6 and ∼8 kDa (Fig. 1B, lane b). Endogenous vesicle hCAP-18/LL-37 was successfully depleted using an anti-LL-37 antibody column (Fig. 1B, lane c). No processing was noted upon incubation with a crude rhCAP-18 peptide (Fig. 1B, lane a), whereas the positive control (a synthetic LL-37 peptide) yielded a 4.5-kDa band (Fig. 1B, lane d).

### Endogenous hCAP-18/LL-37 directly contributes to upregulation of IL-8 mRNA

After depleting endogenous hCAP-18/LL-37 via specific antibody column purification, LSEs were incubated with the depleted PPP-VF using the method described above. The IL-8 mRNA level upon stimulation with depleted PPP-VF was significantly lower than that after incubation with non-depleted PPP-VF (Fig. 1C). However, no significant differences in the levels of IL-17C, IL-1α, or IL-1β mRNAs were evident (Fig. 1C).

### PPP-VF contains a higher concentration of hCAP-18/LL-37 than it in eccrine sweat

**Figure 2:**
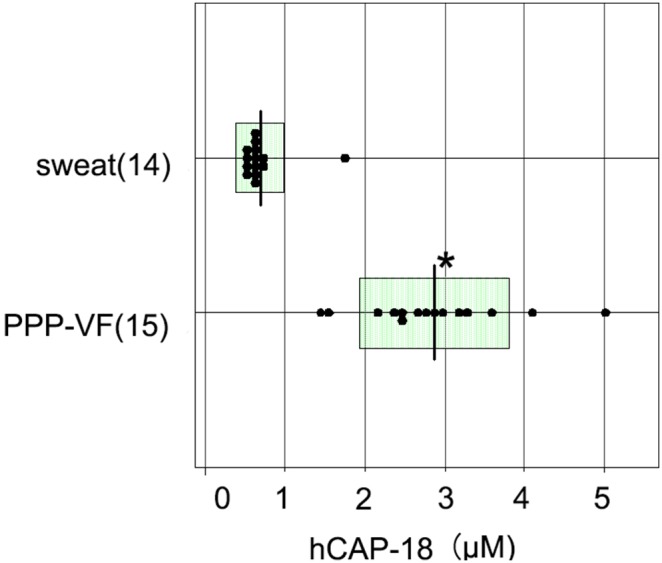
Quantification of hCAP-18/LL-37 in PPP-VF and eccrine sweat samples. Dot-blot analyses and densitometry were performed on PPP vesicles (15 samples), eccrine sweat samples (14 samples), a serially diluted LL-37 synthetic peptide solution, and a 10 µM solution of scrambled LL-37 synthetic peptide (negative control). hCAP-18/LL-37 was confirmed to be present in all PPP-VF and eccrine sweat samples, but not in the scrambled peptide control. The average concentrations of hCAP-18/LL-37 in PPP-VF and control sweat were 2.87±0.93 and 0.09±0.09 µM, respectively. *p<0.05 compared to sweat.

To determine hCAP-18/LL-37 concentrations in PPP-VF, we performed dot-blot analysis and densitometry using sweat as a control. All 15 PPP-VF samples and all 14 samples of healthy sweat contained hCAP-18/LL-37 (Fig. 2). The average concentration of hCAP-18/LL-37 in PPP-VF was 2.87±0.93 µM, but the average concentration in sweat was only 0.09±0.09 µM, consistent with the results of a previous study reporting an average hCAP-18/LL-37 concentration ∼1 µM in crude sweat collected from normal volunteers [30]. The difference was significant (*p*<0.05).

### LL-37 (the mature form of hCAP-18) upregulates PPP-associated cytokines in NHKs

**Figure 3:**
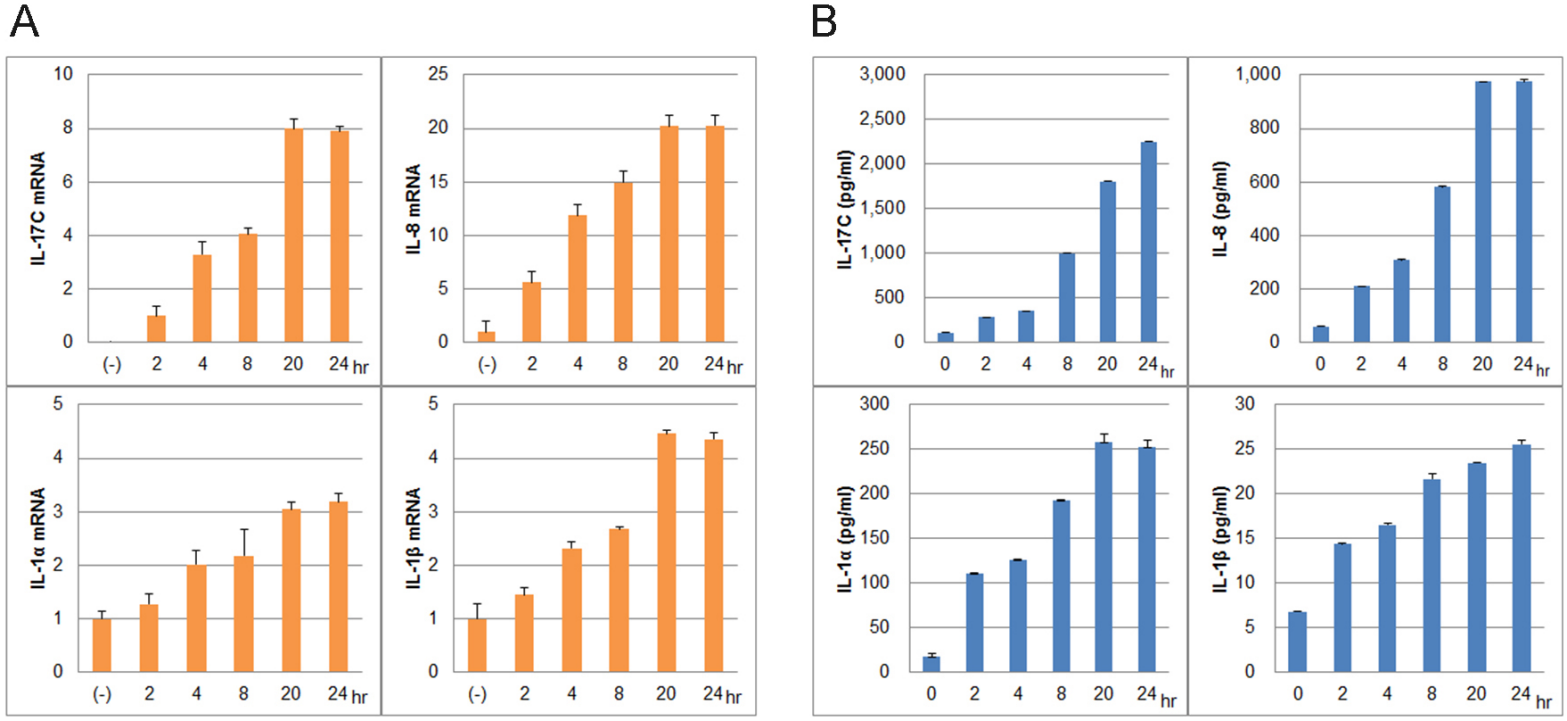
Cytokine induction in NHKs by the synthetic LL-37 peptide. To assess the ability of LL-37 to induce cytokines, NHKs were incubated with 3 µM LL-37 for 0, 2, 4, 8, 20, and 24 h at 37°C. (A) Expression levels of mRNAs encoding IL-17C, IL-8, IL-1α, and IL-1β mRNA, measured by qRT-PCR. The relative mRNA levels are expressed as means ±SDs (in -fold changes). Enzyme-linked immunoassays (ELISAs) were performed on culture media. All later values yielded by both qRT-PCR and ELISA were significantly different from those at 0 h (A, B, *p*<0.05).

As hCAP-18/LL-37 was present in PPP-VF, we next explored whether LL-37 stimulated proinflammatory cytokine expression in NHKs. Pilot experiments indicated that stimulation with 3 µM LL-37 was appropriate. NHKs were incubated with 3 µM synthetic LL-37 for 0, 2, 4, 8, 20 and 24 h at 37°C, and mRNA levels next evaluated via qRT-PCR. LL-37 stimulated upregulation of mRNAs encoding IL-17C, IL-8, IL-1α and IL-1β; but not IL-22, IL-17A, or IL-17F (Fig. 3A). Upregulation of mRNAs encoding IL-8, IL-1α and IL-1β was defined by increases from the values at 0 h. However, IL-17C mRNA was not expressed at 0 h and upregulation was thus defined by reference to the level at 2 h. All mRNA expression levels increased in a time-dependent manner, but to significantly different extents. To confirm protein expression, culture media were assessed by ELISAs (Fig. 3B). IL-17C, IL-8, IL-1α, and IL-1β protein levels in media increased in parallel with the corresponding mRNA levels, suggesting that PPP-VF contained a level of LL-37 sufficient to induce IL-17C, IL-8, IL-1α, and IL-1β mRNA expression in NHKs, consistent with data on mRNA expression levels in PPP lesional skin biopsy samples [32].

### hCAP-18 is processed to LL-37 in PPP-VF

**Figure 4:**
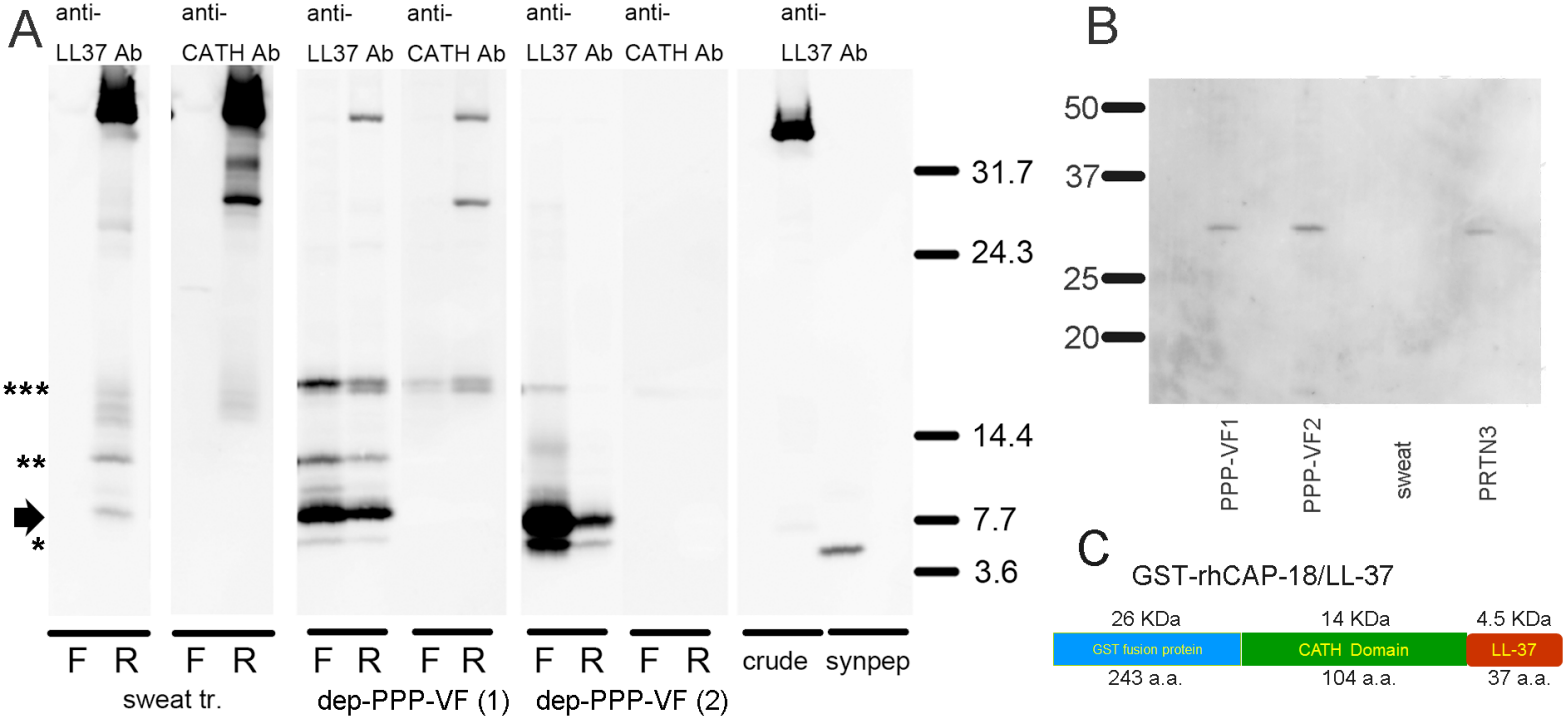
LL-37 is synthesized from GST-rhCAP18 in depleted PPP-VF containing proteinase 3. **A)** Several bands derived from GST-rhCAP18 were evident with dep-PPP-VF incubation. These were hCAP-18 (18 kDa; full length, indicated with ***), an intermediate-sized fragment (∼14 kDa, indicated with **), mature LL-37 (4.5 kDa, indicated with *), and two additional bands of ∼6 and 8 kDa (indicated with right arrow). In addition, 18-kDa bands reacting with anti-CATH Ab were present in PPP-VF tr-1 (F and R) and PPP-VF tr-2 (F). No mature LL-37 was detected in the sweat treated sample (lane R: sweat tr; anti-LL37 Ab staining). Abbreviations: α-LL37, anti-LL-37 antibody; α-CATH, anti-CATH antibody; F, peptide in flowthrough; R, peptide binding to resin; Sweat tr, eccrine sweat-treated peptide; PPP-VF tr, depleted PPP-VF component-treated peptide; crude, non-treated GST-hCAP-18 peptide (binding to resin); syn pep, LL-37 synthetic peptide (3.2 pmol). **B)** Proteinase 3 expression in concentrated PPP-VF was confirmed by Western blotting. Both depleted samples, PPP-VFs 1 and 2, exhibited single bands 29 kDa in size, thus that of PRTN3. Authentic PRTN3 (10 ng) served as a positive control. PPP-VF tr, depleted PPP-VF component-treated peptide; sweat, sweat sample 1; PRTN3, native proteinase 3. **C)** The illustration of structure of GST-rhCAP-18/LL-37.

To determine whether hCAP-18 in PPP-VF could be processed to LL-37 by proteinases in PPP-VF, GST-rhCAP-18 was incubated with dep-PPP-VF from individual two cases described above. Western blotting revealed several processed bands, and the hCAP-18 (18 kDa; full-length) protein. Derived bands included an intermediate-sized fragment (∼14 kDa), mature LL-37 (4.5 kDa), and two additional bands of ∼6 and ∼8 kDa (Fig. 4A, lanes F [flowthrough] and R [resin-bound] material from dep-PPP-VF (1); and in lane F of material from dep-PPP-VF (2); detected using an anti-LL37 Ab). In a separate experiment, we found that GST-rhCAP-18 incubated in eccrine sweat was cleaved into hCAP-18 (18 kDa; full-length) protein, an intermediate-sized fragment (∼14 kDa) and two additional bands of ∼6 and ∼8 kDa, but mature LL-37 (4.5 kDa)was not detected (Fig. 4A, lane R of sweat tr; detected using an anti-LL-37 Ab). In addition, 18-kDa bands were detected using anti-CATH Ab in both dep-PPP-VF (1) (F and R) and dep-PPP-VF (2). These results suggest that PPP-VF contains a proteinase that can process LL-37. However, although sweat also contains proteinases, these cannot directly process hCAP-18 to LL37.

### PPP-VF contains proteinase 3

We used Western blotting to determine whether proteinase 3 [21], which processes LL-37, was present in PPP-VF. A band 29 kDa in size, thus that of native proteinase 3, was present in PPP-VF but not in the control sweat sample (Fig. 4B).

### PPP-VF contains CD68-positive cells

**Figure 5:**
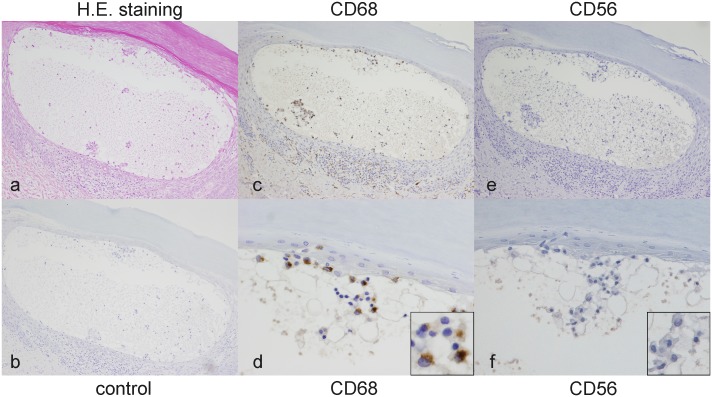
Monocytes in PPP-VF. Hematoxylin-eosin staining revealed many mononuclear cells, but no polymorphonuclear cells, in vesicles (Fig. 5a). The mononuclear cells were positive for CD68 (Figs. 5c, d) but not CD56 (Figs. 5e, f) in all five instances. Pre-immune anti-mouse IgG did not stain the sections. (Fig. 5d). (Original magnifications: a, b, c, e: 100×, d, f: 400×).

H&E staining revealed that vesicle contained many mononuclear cells but no polymorphonuclear cells (Fig. 5a). The mononuclear cells were positive for CD68 (Fig. 5c, d) but not for CD56 (Fig. 5e, f), suggesting that the cells were monocytes/macrophages and not NK or plasma cells. No signal was detected from pre-immune mouse IgG (Fig. 5b).

### Lesion epidermis expresses monocyte chemotactic chemokine (MCP-1) protein

**Figure 6:**
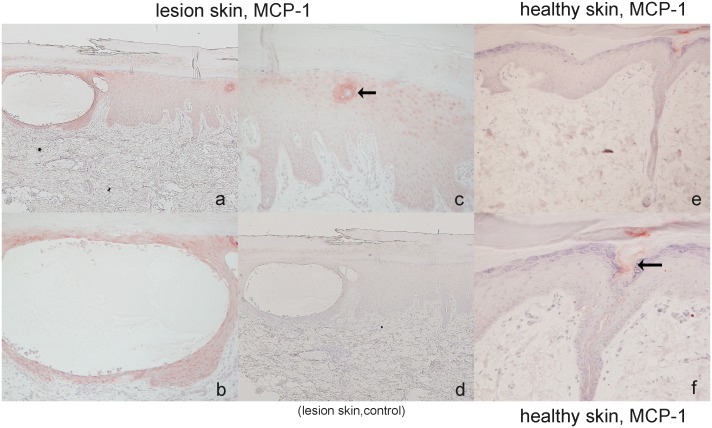
MCP-1 expression in PPP lesion skin and healthy skin. In lesion skin, strong expression of MCP-1 was detected around the PPP vesicle in the epidermis (Figs. 6a, b). In addition, acrosyringium in the lesions skin also showed the protein expression (Fig. 6c), but not in healthy skin (Fig. 6e, f). The eccrine pore at the surface of skin showed weak positive staining locating (Fig. 6f, arrowhead). (Original magnifications: a, c, d, e: 40×, b, f: 100×).

The epidermis surrounding the PPP vesicle expressed the MCP-1 protein (Fig. 6a–c, e, f). No signal was detected from pre-immune mouse IgG (Fig. 6d).

## Discussion

Previously, we reported that IL-17 (A, C, and F), IL-22, and IL-8 mRNAs were upregulated in the lesional skin of PPP patients [32]; confirmed that PPP-VF could upregulate the expression of IL-17C, IL-8, IL-1α, and IL-1β in LSE keratinocytes; and that endogenous hCAP-18/LL-37 induced high-level IL-8 synthesis (Fig. 1). PPP-VF contains a higher concentration of hCAP-18/LL-37 than does eccrine sweat (Fig. 2), sufficient to induce IL-17C, IL-8, IL-1α, and IL-1β mRNA and protein expression in monolayer NHKs (Fig. 3). In addition, GST-rhCAP-18 was incubated with depleted PPP-VF, and the presence of processed LL-37 confirmed thereafter (Fig. 4A). This suggests that PPP-VF contains the proteinase responsible for cleavage of hCAP-18 to LL-37, and, indeed, Western blotting confirmed that proteinase 3 was present in PPP-VF (Fig. 4B).

This is the first report to show that PPP-VF induces IL-17C, IL-8, IL-1α, and IL-1β synthesis in keratinocytes of LSEs, and to quantify the hCAP-18/LL-37 concentration in PPP-VF. The average concentration of LL-37 in PPP-VF was 2.87±0.93 µM, and this was sufficient to induce IL-17C, IL-8, IL-1α, and IL-1β mRNA and protein expression in monolayer NHKs.

We have shown that 3 µM LL-37 was sufficient to stimulate IL-8 release from cultured keratinocytes, without toxic effects [22], [24], and that LL-37 at 13.5 or 45 µg/ml (3 or 10 µM, respectively) increased production of IL-6 and secretion of IL-1α [22]. LL-37 enhanced IL-8 release by human airway smooth muscle (HASM) cells, via a process dependent on ERK1/2 activation [23]. IL-17 also induced release of IL-8 and IL-6 from human keratinocytes, but the amounts released were lower than noted upon LL-37 induction. However, the Th17 cytokines (IL-17 and IL-22) synergistically upregulated IL-8 and IL-6 expression in keratinocytes [25]. Upon PPP-VF stimulation in LSE, we could not confirm IL-22 upregulation in keratinocytes, but IL-17C upregulation was indeed evident. However, the level of IL-17C mRNA in LSE was not greatly reduced in dep-PPP-VF. The big difference between monolayer culture cells and LSE is that LSE has piled-up keratinocytes covered with stratum corneum and also has a lot of differentiated keratinocyte such as granular cells and prickle cells. Though LSE was stimulated with LL-37 through the stratum corneum with a gel cylinder, NHK were stimulated with LL-37 in the culture medium which they were covered. Under inflammatory skin condition, stratum corneum could be easily disrupted so that keratinocyte could not have such a protection no more. In addition, from the view of cell differentiation, many of keratinocytes in monolayer culture might be in proliferative stage, but it in LSE might be constructed with many differentiated keratinocytes after air-lifting. We are speculating that these differences are responsible, so far. However the detail of the reason is still unclear so that we are now continuing to elucidate this reason from the view of the skin barrier problem and keratinocyte differentiation.

We earlier reported that PPP-VF was formed in the acrosyringium, and thus originally contains components of eccrine sweat [2], and sweat contains IL-1α and IL-1β [33]. Thus, baseline IL-1α and IL-1β mRNA levels reflect not only the action of the endogenous hCAP-18/LL-37 system but also the fact that sweat components are included in vesicles. We have also reported that sweat IL-1α induced IL-8 expression in keratinocytes [33]. This may explain why baseline IL-8 mRNA expression remained high even after endogenous hCAP18/LL-37 was removed from PPP-VF.

From the result of LSE stimulation with dep-PPP-VF experiment, IL-8 mRNA expression was significantly decreased in dep-PPP-VF. This suggests that IL-8 expression in keratinocytes is directly upregulated by LL-37, consistent with our previous data showing that LL-37 potently stimulated IL-8 release from keratinocytes [24]. IL-8 is regarded as a major factor triggering pustule formation [7]. IL-8 action has been implicated in a number of inflammatory diseases involving neutrophil activation. A human mAb directed against IL-8 (HuMab 10F8), which neutralizes IL-8-dependent human neutrophil activation and migration, has been evaluated (in a Scandinavian Phase 2 trial) as a candidate for PPP treatment [34]. This trial was well-tolerated and significantly reduced clinical disease activity at all five endpoints evaluated; fresh pustule formation was reduced by ≥50%. This result strongly suggests that IL-8 is involved in the pathogenesis of PPP pustulation. IL-8 is barely detectable in healthy skin, but it is rapidly induced (by 10–100-fold) in response to several proinflammatory cytokines (including tumor necrosis factor and IL-1), bacterial and viral products, cellular stress [35], and IL-17 [36]. As mentioned above, we confirmed that the average concentration of hCAP-18/LL-37 was 2.87±0.93 µM, which can directly induce expression of both IL-8 mRNA and protein. This suggests that IL-8 in keratinocytes was induced principally via LL-37 stimulation. However, it has been reported that several factors, including IL-17A/C/F, IL-1α, and IL-1β, can upregulate IL-8 expression in keratinocytes. The mechanism of IL-8 upregulation in PPP requires further elucidation, but it is clear that LL-37 may play a role and may also contribute to subsequent inflammation of PPP lesional skin.

LL-37, mature form of human cathelicidin, can be processed after secretion in sweat physiologically, and fragments derived from LL-37, including RK-31, KR-30, and KS-20, exhibit antimicrobial activity as LL-37 shows [24]. Additional LL-37 fragments are found in the pathogenesis of several inflammatory skin disorder, such as rosacea, and they contribute the inflammatory cytokines up-regulations [25]. Hence, we speculated that fragments derived from LL-37 might be processed in early stage PPP vesicles which could trigger subsequent inflammation. However, western blotting revealed no fragment smaller than LL-37 in PPP-VF.

PPP vesicle originally contains additional 6 and 8 kDa in addition to 18 and 4.5 kDa (figure 1B, lane b), and GST-rhCAP-18 treated with dep-PPP-VF showed all of them and another band about 10 kDa (Fig. 4 A). With sweat treatment, those bands were detected without 4.5 kDa. This result suggested that not only the proteinase for LL-37 processing was included but also other proteinase which could process or degrade the GST-rhCAP-18 protein. And it was also suggested that control sweat we collected did not contain enough concentration of the responsible proteinase, such as proteinase 3 [21], to process LL-37.

The three known serine proteases in azurophil granules (elastase, cathepsin G, and proteinase 3) cleave many of the same substrates, and hCAP-18 is susceptible to cleavage by all three enzymes in vitro. However, proteinase 3 is solely responsible for cleavage of hCAP-18 after exocytosis [21]. Proteinase 3 is a 29-kDa serine protease normally transcribed during myelopoiesis, and is thought to be turned off in both mature leucocytes [21], [37] and monocytes [38]–[40]. We thus doubted that PPP-VF contained proteinase 3, and Western blotting showed that the proteinase was indeed present.

We very carefully collected PPP-VF using a dermoscope not to collect polymorphonuclear cells such as neutrophils. When infiltrating cells were evaluated histopathologically, early stage vesicles contained principally mononuclear cells suggestive of monocytes/macrophages with only a few polymorphic nuclear cells. Upon immunostaining for CD68, many vesicular cells were positive, especially near the lids of vesicles, suggesting that proteinase 3 in early stage vesicles is secreted from monocytes that had infiltrated the vesicles. For further study to know if the epidermis expressed CCL2 expression, MCP-1 immunohistochemistry was additionally performed because CCL2 is a strong monocyte/macrophage chemoattractant. The epidermal keratinocyte surrounding PPP vesicle showed MCP-1 protein expression with immunohistochemical examination. The recruitment of CD68 positive mononuclear cells in the early vesicles could be induced by this phenomenon (Fig. 5). However, if so, the exact mechanism by which monocytes/macrophages infiltrate the PPP-VF in the early stages of disease remains still unclear, and need to elucidate further.

In the present study, we thus report a new role for the LL-37 of PPP-VF. The protein contributes to the pathophysiology of subsequent inflammation. However, further studies are required to clarify how PPP is initiated.
